# Protein structural similarity search by Ramachandran codes

**DOI:** 10.1186/1471-2105-8-307

**Published:** 2007-08-23

**Authors:** Wei-Cheng Lo, Po-Jung Huang, Chih-Hung Chang, Ping-Chiang Lyu

**Affiliations:** 1 Institute of Bioinformatics and Structural Biology, National Tsing Hua University, 101, Section 2 Kuang Fu Road, Hsinchu 30013, Taiwan

## Abstract

**Background:**

Protein structural data has increased exponentially, such that fast and accurate tools are necessary to access structure similarity search. To improve the search speed, several methods have been designed to reduce three-dimensional protein structures to one-dimensional text strings that are then analyzed by traditional sequence alignment methods; however, the accuracy is usually sacrificed and the speed is still unable to match sequence similarity search tools. Here, we aimed to improve the linear encoding methodology and develop efficient search tools that can rapidly retrieve structural homologs from large protein databases.

**Results:**

We propose a new linear encoding method, SARST (Structural similarity search Aided by Ramachandran Sequential Transformation). SARST transforms protein structures into text strings through a Ramachandran map organized by nearest-neighbor clustering and uses a regenerative approach to produce substitution matrices. Then, classical sequence similarity search methods can be applied to the structural similarity search. Its accuracy is similar to Combinatorial Extension (CE) and works over 243,000 times faster, searching 34,000 proteins in 0.34 sec with a 3.2-GHz CPU. SARST provides statistically meaningful expectation values to assess the retrieved information. It has been implemented into a web service and a stand-alone Java program that is able to run on many different platforms.

**Conclusion:**

As a database search method, SARST can rapidly distinguish high from low similarities and efficiently retrieve homologous structures. It demonstrates that the easily accessible linear encoding methodology has the potential to serve as a foundation for efficient protein structural similarity search tools. These search tools are supposed applicable to automated and high-throughput functional annotations or predictions for the ever increasing number of published protein structures in this post-genomic era.

## Background

The number of proteins found in structural databases has increased at such an unprecedented rate in recent years that achieving speed and accuracy simultaneously in protein structure similarity searches has become a formidable task. During evolution, three-dimensional (3D) structures are more conserved than amino acid sequences [1], and protein homologs that share highly conserved 3D structures may have unrecognizable sequence homology [2]. Amino acid sequence search tools are fast; however, they have proven to be insufficient for detection of remote homology in structural databases [3]. Structure alignment using delicate geometric algorithms is much more accurate than amino acid sequence comparisons, especially when the sequence homology is low [3]. Many brilliant pairwise comparison tools have been created, such as Distance Alignment Tools, DALI [4], Combinatorial Extension, CE [5], and FAST Alignment and Search Tool, FAST [6], but still there is a demand for rapid similarity search tools because protein structure databases have outgrown the utility of pairwise-based searches.

Protein structures are not fully flexible; there are physical constraints on polypeptide conformation [7-11]. It is believed that the 3D structure can be reduced to a simpler form while maintaining the intrinsic structural information [12-24]. With the reduced data, a similarity search can become much easier and faster. A number of methods have been designed based on this idea to one-dimensionalize the 3D protein structure. For instance, Levine et al. (1984) compared 3D protein structures using the sequence of dihedral angles (*ϕ*, *ψ*) in a pairwise manner [14]. Lesk (1998) modified Efimov's dissections on the Ramachandran diagram [15] and combined them with a reduced amino acid alphabet to linearly encode protein structures [16]. Martin (2000) developed TOPSCAN, which uses topology strings to represent protein structures [17]. However, most of these methods could not reach the accuracy comparable to conventional 3D structural comparison methods, and furthermore the implementation of some of them were limited because their methodology could not conveniently analyze fragments with missing residues [18]. Consequently, linear encoding methods have long been considered to compromise accuracy for speed in protein structure comparisons [14]. Nevertheless, there are advantages of the one-dimensional (1D) representation of protein structure, such as its easy applicability to multiple structural alignments [16], fold-recognition and genome annotations [19,20]; besides, local backbone structure prediction has long been using linear encoding methodologies [24-27].

In recent years, linear encoding has been applied to large scale structural database searches. Methods like YAKUSA [21] and 3D-BLAST [22] can scan thousands of proteins thousands of times as rapid as CE with good performance in searching accuracy. In this post-genomic era when protein structural data increase exponentially, we believe that linear encoding methodology is capable of serving as the foundation for efficient protein structural similarity search tools and that there is still much valuable room left for its improvement.

**Figure 1:**
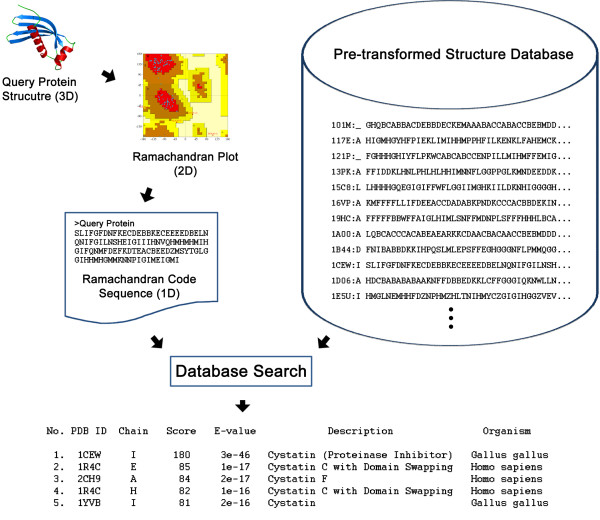
**Flowchart of SARST approaches**. Three-dimensional (3D) protein structures are first transformed onto two-dimensional (2D) Ramachandran maps and then further converted into one-dimensional (1D) text strings. Thus, a structural similarity search could be performed by classical sequence similarity search methods. The Ramachandran plot shown here was generated by PROCHECK [54]. (Note that "similarity search" is more typically termed as "alignment search"; however, considering that SARST is designed as a search method rather than an alignment tool, we will use the former term throughout this report to avoid misunderstanding.)

Here we propose a linear encoding algorithm, Ramachandran sequential transformation, and introduce an efficient protein structural similarity search method, SARST (Structural similarity search Aided by Ramachandran Sequential Transformation) [28]. SARST improves the linear encoding methodology and achieves higher search speed with less sacrifice of accuracy than previous methods. SARST converts 3D protein structures into two-dimensional Ramachandran maps [29] and further to 1D sequences by predefined assignments of regions to text letters (Ramachandran codes). Finally, conventional sequence similarity search methods can be applied to retrieve homologous proteins from structure databases. These approaches are illustrated in Figure 1.

SARST, using structurally meaningful Ramachandran strings, converts structural similarity search problems into sequence similarity searches. Besides inheriting the speed advantages of sequence-based methods, it provides a ranked hit list with similarity scores and statistically meaningful expectation values (E-values) to assess the reliability of the retrieved information.

Because SARST is aimed to be a database search method, information retrieval (IR) techniques, which have been widely used in the document, image, spatial database, and 3D protein structure database fields [30,31], were used to evaluate it. SARST can detect remote homology and overcome structural incompleteness; we also report its performances on different structural classes (all alpha, all beta, alpha/beta and alpha+beta).

## Results

### Algorithm – Ramachandran sequential transformation (RST)

One thousand domains, each composed of a single polypeptide chain without missing residues, were randomly selected as the training set from the ASTRAL SCOP 1.67 40% identities (ID) subset [32-34] [see Additional file 1]. For every residue *r*_*n *_in the training set, the torsion angle *phi *(*ϕ*) formed by atoms of *r*_*n*-1 _and *r*_*n*_, and *psi *(*ψ*) formed by *r*_*n *_and *r*_*n*+1 _were calculated to convey the two-residue-long backbone conformation involving three consecutive residues. All the torsion angle pairs (*ϕ*, *ψ*) were mapped onto the Ramachandran (RM) plot, and their distribution was analyzed by counting the pairs contained in each 10° × 10° unit cell. There were 36 × 36 = 1,296 cells on the RM map, each with a known number of (*ϕ*, *ψ*) spots. These cells were clustered into 22 groups based on a parameter, *RSAD *(Root Square Angular Distance), defined to represent the "distances" among cells:

RSAD=Sc(Δϕ)2+(Δψ)2
 MathType@MTEF@5@5@+=feaafiart1ev1aaatCvAUfKttLearuWrP9MDH5MBPbIqV92AaeXatLxBI9gBaebbnrfifHhDYfgasaacH8akY=wiFfYdH8Gipec8Eeeu0xXdbba9frFj0=OqFfea0dXdd9vqai=hGuQ8kuc9pgc9s8qqaq=dirpe0xb9q8qiLsFr0=vr0=vr0dc8meaabaqaciaacaGaaeqabaqabeGadaaakeaacqWGsbGucqWGtbWucqWGbbqqcqWGebarcqGH9aqpcqWGtbWudaWgaaWcbaGaem4yamgabeaakmaakaaabaGaeeikaGIaeuiLdqecciGae8x1dOMaeeykaKYaaWbaaSqabeaacqqGYaGmaaGccqGHRaWkcqqGOaakcqqHuoarcqWFipqEcqqGPaqkdaahaaWcbeqaaiabbkdaYaaaaeqaaaaa@41D2@

where -180° < Δ*ϕ *< 180° and -180° < Δ*ψ *< 180°. They represent the differences in *ϕ *and *ψ *angles between a pair of cells. *S*_*c *_is a scaling constant assisting in restricting the number of clustered groups.

The 1,296 cells were first ranked in descending order by their spot numbers and assigned as x_1_–x_1296_; then, each cell was assigned a representative angle pair (*ϕ*_*i*_, *ψ*_*i*_), where *ϕ*_*i *_and *ψ*_*i *_stood for the central *ϕ *and *ψ *angles of x_*i*_, respectively. We defined *n*_*i *_as the spot number of x_*i *_and set up a distance matrix D by assigning each element, D_*ij*_, the *RSAD *between x_*i *_and x_*j*_. With this matrix, a nearest-neighbor clustering algorithm [35] was performed following the steps below:

(1) Set x_*i *_= x_1 _and *g *= 1.

(2) Assign x_*i *_to cluster C_*g *_and let it be the center of C_*g*_. Now, *N*_*g*_, the total number of spots in C_*g*_, is *n*_*i*_.

(3) Find the nearest neighbor of x_*i*_. Let x_*j *_denote it.

(4) For those x_*j *_with *n*_*j *_> 3:

a. If D_*ij *_is smaller than *T*_*D*_, the threshold of distance, *N*_*g *_+ *n*_*j *_is smaller than *T*_*N*_, the threshold of spot number, and x_*j *_has not been clustered, then assign x_*j *_to C_*g*_.

b. If D_*ij *_<*T*_*D*_, *N*_*g *_+ *n*_*j *_<*T*_*N*_, and x_*j *_has been clustered in C_*m*_, compare D_*ij *_and the distance between x_*j *_and the center of C_*m*_. If D_*ij *_is the smaller, reassign x_*j *_to C_*g*_.

(5) For those x_*j *_with *n*_*j *_≤ 3: if D_*ij *_< 0.5 × *T*_*D*_, assign x_*j *_to C_*g*_; otherwise, simply assign it to C_22_.

(6) Repeat steps (4)–(5) with the next nearest neighbor while D_*ij *_<*T*_*D*_.

(7) If every cell has been clustered, then stop. Otherwise, find the cell possessing the most spots from those not yet clustered, let it be the new x_*i *_and set *g *= 2, then go to step (2).

**Figure 2:**
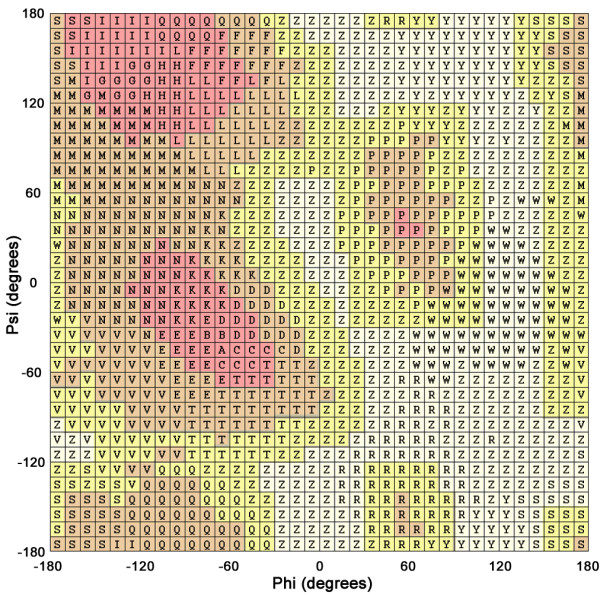
**The Ramachandran map of SARST**. The 1,296 cells on the map were clustered into 22 groups by the Ramachandran sequential transformation algorithm. See text for details.

In this procedure, we were able to adjust *T*_*D*_, *T*_*N *_and *S*_*c *_in formula (1) so as to cluster all the cells into 22 groups. Finally, each group was assigned an English letter. As shown in Figure 2, these assigned letters represented specific regions of the Ramachandran map, and were called "Ramachandran codes". According to these codes, the coordinates of a protein could be transformed into a text sequence in the order of residue serial numbers. If a chain contained missing or internal (*ϕ*, *ψ*) incalculable residues, those positions would be labelled as "X"s. The "sequence" generated by RST algorithm is structurally meaningful and very different from the amino acid sequence in nature; therefore, we call it Ramachandran sequence or Ramachandran string.

### Building scoring matrices – a regenerative approach

Because RM codes differ from amino acids, suitable scoring matrices were created to perform RM sequence alignment searches. We developed a "regenerative approach", which started with a primitive (and trial) matrix and enabled us to produce scoring matrices generation after generation until the quality was acceptable:

**(1) **The densest cell of each RM code region has been assigned as the representative center during RST. Code regions with smaller *RSAD *would be spatially close on the map, which we believed could be given higher scores. Based on this concept, we first calculated the average *RSAD *(RSAD¯
 MathType@MTEF@5@5@+=feaafiart1ev1aaatCvAUfKttLearuWrP9MDH5MBPbIqV92AaeXatLxBI9gBaebbnrfifHhDYfgasaacH8akY=wiFfYdH8Gipec8Eeeu0xXdbba9frFj0=OqFfea0dXdd9vqai=hGuQ8kuc9pgc9s8qqaq=dirpe0xb9q8qiLsFr0=vr0=vr0dc8meaabaqaciaacaGaaeqabaqabeGadaaakeaadaqdaaqaaiabdkfasjabdofatjabdgeabjabdseaebaaaaa@3135@) of all the representative centers and then built the "primitive scoring matrix I" (PSMI) using the formula:

Scoreij=5×−log⁡(RSADij0.5×RSAD¯)for i≠j
 MathType@MTEF@5@5@+=feaafiart1ev1aaatCvAUfKttLearuWrP9MDH5MBPbIqV92AaeXatLxBI9gBaebbnrfifHhDYfgasaacH8akY=wiFfYdH8Gipec8Eeeu0xXdbba9frFj0=OqFfea0dXdd9vqai=hGuQ8kuc9pgc9s8qqaq=dirpe0xb9q8qiLsFr0=vr0=vr0dc8meaabaqaciaacaGaaeqabaqabeGadaaakeaafaqabeqacaaabaGaem4uamLaem4yamMaem4Ba8MaemOCaiNaemyzau2aaSbaaSqaaiabdMgaPjabdQgaQbqabaGccqGH9aqpcqaI1aqncqGHxdaTcqGHsislcyGGSbaBcqGGVbWBcqGGNbWzcqGGOaakdaWcaaqaaiabdkfasjabdofatjabdgeabjabdseaenaaBaaaleaacqWGPbqAcqWGQbGAaeqaaaGcbaGaeGimaaJaeiOla4IaeGynauJaey41aq7aa0aaaeaacqWGsbGucqWGtbWucqWGbbqqcqWGebaraaaaaiabcMcaPaqaaiabbAgaMjabb+gaVjabbkhaYjabbccaGiabdMgaPjabgcMi5kabdQgaQbaaaaa@5B67@

for *i *= *j*, the scores were uniformly appointed as 10.

**(2) **Using PSMI, all-against-all RM sequence alignments were performed between the training set and the ASTRAL SCOP 1.67 50% ID subset by blast [36,37]. FAST [6] was used as a filter to pick the pairs with alignment lengths larger than 50% to form a "primitive pair database".

**(3) **The algorithm of BLOSUM matrices [38] was implemented to this pair database to build primitive scoring matrix II (PSMII).

**(4) **After performing recursive all-against-all RM sequence alignment on the ASTRAL SCOP 1.67 50% ID subset using PSMII, the pairs with FAST alignment lengths larger than 80% were picked to form a "50% ID primitive pair database", which then generated PSMIII.

**(5) **With PSMIII, we repeated step (4) for ASTRAL SCOP 1.67 10, 20, 30, 40, 50, 70, 90, and 100% ID subsets and produced the SARST Scoring Matrix (SARSTSM) 10, 20, 30, 40, 50, 70, 90 and 100, respectively.

### Optimization of the scoring matrix

#### A. Selection of the scoring matrix

In 2004, Aung collected 34,055 proteins covering about 90% of the ASTRAL SCOP 1.59 dataset to form a large target database, from which 108 query proteins were selected [30]. To assess the applicability of the SARSTSM, we adopted this database as well as the following parameters commonly used in the information retrieval experiments:

Recall=Number of relevant retrievalsTotal number of relevant proteins
 MathType@MTEF@5@5@+=feaafiart1ev1aaatCvAUfKttLearuWrP9MDH5MBPbIqV92AaeXatLxBI9gBaebbnrfifHhDYfgasaacH8akY=wiFfYdH8Gipec8Eeeu0xXdbba9frFj0=OqFfea0dXdd9vqai=hGuQ8kuc9pgc9s8qqaq=dirpe0xb9q8qiLsFr0=vr0=vr0dc8meaabaqaciaacaGaaeqabaqabeGadaaakeaacqqGsbGucqqGLbqzcqqGJbWycqqGHbqycqqGSbaBcqqGSbaBcqGH9aqpdaWcaaqaaiabb6eaojabbwha1jabb2gaTjabbkgaIjabbwgaLjabbkhaYjabbccaGiabb+gaVjabbAgaMjabbccaGiabbkhaYjabbwgaLjabbYgaSjabbwgaLjabbAha2jabbggaHjabb6gaUjabbsha0jabbccaGiabbkhaYjabbwgaLjabbsha0jabbkhaYjabbMgaPjabbwgaLjabbAha2jabbggaHjabbYgaSjabbohaZbqaaiabbsfaujabb+gaVjabbsha0jabbggaHjabbYgaSjabbccaGiabb6gaUjabbwha1jabb2gaTjabbkgaIjabbwgaLjabbkhaYjabbccaGiabb+gaVjabbAgaMjabbccaGiabbkhaYjabbwgaLjabbYgaSjabbwgaLjabbAha2jabbggaHjabb6gaUjabbsha0jabbccaGiabbchaWjabbkhaYjabb+gaVjabbsha0jabbwgaLjabbMgaPjabb6gaUjabbohaZbaaaaa@8632@

Precision=Number of relevant retrievalsTotal number of retrieved proteins
 MathType@MTEF@5@5@+=feaafiart1ev1aaatCvAUfKttLearuWrP9MDH5MBPbIqV92AaeXatLxBI9gBaebbnrfifHhDYfgasaacH8akY=wiFfYdH8Gipec8Eeeu0xXdbba9frFj0=OqFfea0dXdd9vqai=hGuQ8kuc9pgc9s8qqaq=dirpe0xb9q8qiLsFr0=vr0=vr0dc8meaabaqaciaacaGaaeqabaqabeGadaaakeaacqqGqbaucqqGYbGCcqqGLbqzcqqGJbWycqqGPbqAcqqGZbWCcqqGPbqAcqqGVbWBcqqGUbGBcqGH9aqpdaWcaaqaaiabb6eaojabbwha1jabb2gaTjabbkgaIjabbwgaLjabbkhaYjabbccaGiabb+gaVjabbAgaMjabbccaGiabbkhaYjabbwgaLjabbYgaSjabbwgaLjabbAha2jabbggaHjabb6gaUjabbsha0jabbccaGiabbkhaYjabbwgaLjabbsha0jabbkhaYjabbMgaPjabbwgaLjabbAha2jabbggaHjabbYgaSjabbohaZbqaaiabbsfaujabb+gaVjabbsha0jabbggaHjabbYgaSjabbccaGiabb6gaUjabbwha1jabb2gaTjabbkgaIjabbwgaLjabbkhaYjabbccaGiabb+gaVjabbAgaMjabbccaGiabbkhaYjabbwgaLjabbsha0jabbkhaYjabbMgaPjabbwgaLjabbAha2jabbwgaLjabbsgaKjabbccaGiabbchaWjabbkhaYjabb+gaVjabbsha0jabbwgaLjabbMgaPjabb6gaUjabbohaZbaaaaa@8BD2@

A protein is regarded as "relevant" if it belongs to the same SCOP family classification as the query. These two parameters always had opposite tendencies; when attempts were made to ask for higher recalls with the same query, the precision would decrease. Because of this property, to judge the quality of IR experiments, the F-measure [39] was also used:

F = 2 × Recall × Precision/(Recall + Precision)

For every query protein, RM sequence searches were performed asking for 50 to 5,000 retrievals. We observed that SATSTSM20 outperformed other matrices in most of the cases [see Additional file 2].

#### B. Determination of the score scaling factor

BLOSUM matrices were generated using Henikoffs' formula [38]:

*Score*_*ij *_= *f*_*s *_× log_2_(*q_ij_/e_ij_*)

where *q*_*ij *_is the observed and *e*_*ij *_is the expected probability of the occurrence for each *i*, *j *pair, and *f*_*s *_is a scaling factor. In their study, *f*_*s *_was appointed as 2. To optimize our scoring matrix, we selected the "20% ID pair database", which produced SARSTSM20, and then adjusted the scaling factor. The highest average F-measure (70.0%) after the retrieval of 500 proteins was determined with *f*_*s *_= 1.78. Accordingly, the matrix produced from the 20% ID pair database with *f*_*s *_= 1.78 was chosen as the standard scoring matrix for SARST (Table 1).

**Table 1:** The standard scoring matrix of SARST

	A	B	C	D	E	T	K	V	N	F	G	H	I	L	M	Q	S	Y	R	P	W	Z	X
A	3	2	2	1	1	0	-2	-3	-3	-8	-11	-11	-13	-8	-8	-9	-14	-9	-7	-8	-7	-4	0
B	2	2	2	1	1	1	0	-1	-2	-6	-12	-10	-10	-7	-7	-6	-10	-8	-5	-6	-4	-6	0
C	2	2	2	1	1	3	-1	-2	-3	-6	-13	-11	-9	-7	-8	-7	-9	-10	-2	-7	-5	-3	0
D	1	1	1	3	1	2	2	-1	-1	-4	-9	-7	-8	-4	-6	-5	-7	-4	1	-3	-4	-2	0
E	1	1	1	1	3	1	2	3	1	-5	-7	-6	-7	-4	-4	-4	-7	-2	-1	-5	-3	-1	0
T	0	1	3	2	1	5	-1	2	-1	-2	-6	-6	-4	-4	-5	-2	-4	-4	2	-1	-1	3	0
K	-2	0	-1	2	2	-1	4	1	3	-3	-6	-6	-5	-3	-3	-2	-5	-2	-2	0	0	-1	0
V	-3	-1	-2	-1	3	2	1	9	3	-3	-4	-4	-2	-2	-2	0	0	3	2	-1	3	4	0
N	-3	-2	-3	-1	1	-1	3	3	5	-2	-4	-4	-3	-2	0	-2	-3	-2	-1	1	1	1	0
F	-8	-6	-6	-4	-5	-2	-3	-3	-2	5	-1	1	0	3	0	3	0	2	0	-2	-2	1	0
G	-11	-12	-13	-9	-7	-6	-6	-4	-4	-1	4	3	3	0	2	0	1	-3	-5	-5	-6	-2	0
H	-11	-10	-11	-7	-6	-6	-6	-4	-4	1	3	4	1	2	2	0	-1	-2	-4	-3	-5	-1	0
I	-13	-10	-9	-8	-7	-4	-5	-2	-3	0	3	1	4	0	1	2	4	0	-1	-4	-7	-2	0
L	-8	-7	-7	-4	-4	-4	-3	-2	-2	3	0	2	0	4	1	1	-1	0	0	-1	-2	1	0
M	-8	-7	-8	-6	-4	-5	-3	-2	0	0	2	2	1	1	4	0	1	-1	-4	-2	-2	1	0
Q	-9	-6	-7	-5	-4	-2	-2	0	-2	3	0	0	2	1	0	6	1	3	1	-3	-3	1	0
S	-14	-10	-9	-7	-7	-4	-5	0	-3	0	1	-1	4	-1	1	1	7	5	2	-3	-3	3	0
Y	-9	-8	-10	-4	-2	-4	-2	3	-2	2	-3	-2	0	0	-1	3	5	10	7	2	2	7	0
R	-7	-5	-2	1	-1	2	-2	2	-1	0	-5	-4	-1	0	-4	1	2	7	11	3	0	7	0
P	-8	-6	-7	-3	-5	-1	0	-1	1	-2	-5	-3	-4	-1	-2	-3	-3	2	3	8	7	4	0
W	-7	-4	-5	-4	-3	-1	0	3	1	-2	-6	-5	-7	-2	-2	-3	-3	2	0	7	9	5	0
Z	-4	-6	-3	-2	-1	3	-1	4	1	1	-2	-1	-2	1	1	1	3	7	7	4	5	6	0
X	0	0	0	0	0	0	0	0	0	0	0	0	0	0	0	0	0	0	0	0	0	0	0

#### C. Determination of the X scores

In RM strings, code "X"s stood for missing residues, residues with incomplete backbone coordinates, or those providing insufficient information for the calculation of torsion angles. We supposed the X scores should be zero to exert a minimum effect on the accuracy of SARST. After the retrieval of 500 proteins with integer X score ranging from -3 to 3, the highest average F-measures (70.0%) was found at zero X score, in agreement with our supposition.

### Evaluation of speed

Aung and Tan have used their large database to assess the performances of ProtDex2 and several other methods [30]. We adopted their system and added our assessments to CE, FAST, YAKUSA [21], 3D-BLAST [22], BLAST [37] and SARST.

As shown in Table 2, when using a single 3.2-GHz CPU to search this large database (34,055 proteins), SARST registered an average running time of 0.34 second, almost as rapid as BLAST (0.30 second). The SARST running time is approximately 243,500, 18,400, 250, 105, 27 and over 2 times faster than CE, FAST, TOPSCAN, YAKUSA, 3D-BLAST and ProtDex2, respectively. In a multi-processor system, SARST is capable of distributing the calculation work. If dual 3.2-GHz hyperthreading processors were used, its average running time would be 0.16 second, about 5 times faster than ProtDex2 and 517,400 times faster than CE, which itself could not recruit multiple processors.

**Table 2:** Speed comparisons for 108 queries on a database of 34055 proteins

Method	Average time per query (sec)	Average time per comparison (sec)	Relative to SARST
CE	82789.20	2.43E+00	243497.65
FAST	6241.57	1.83E-01	18357.56
TOPSCAN^a^	85.08	2.50E-03	250.24
YAKUSA	35.6	1.05E-03	104.71
3D-BLAST	9.07	2.66E-04	26.68
ProtDex2	0.76	2.23E-05	2.24
BLAST	0.30	8.76E-06	0.88
SARST	0.34	9.98E-06	1.00
SARST (2 CPUs^b^)	0.16	4.70E-06	0.47

^a^These results were adapted from Aung and Tan's report [30].

^b^Two hyperthreading 3.2 GHz CPUs were used to obtain these results, others were calculated using one

### Evaluation of accuracy

**Figure 3:**
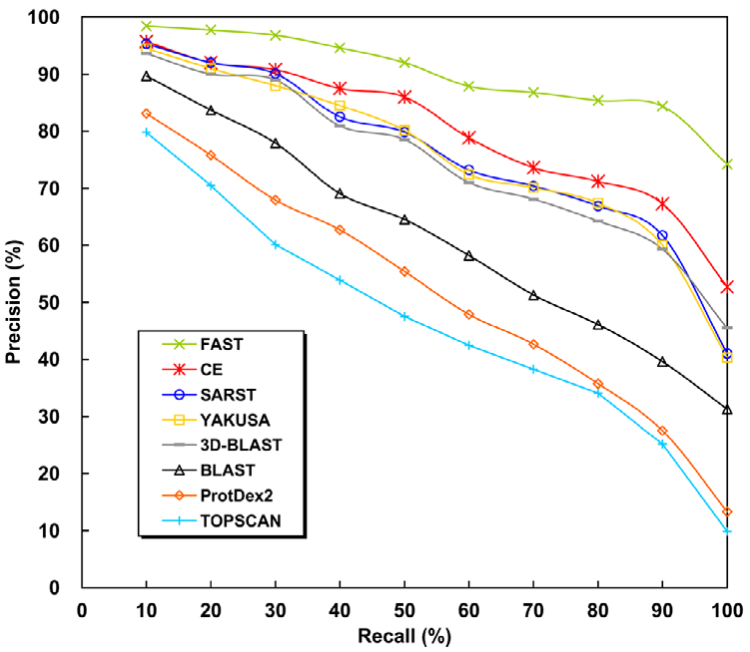
**Average precision-recall curves of several search methods**. FAST was the most accurate search method. SARST ranked third and achieved precisions ~4% lower than CE, which was the second most accurate method in this experiment. Linear encoding methods TOPSCAN [17], YAKUSA [21] and 3D-BLAST [22] describe protein structures as strings. ProtDex2 transforms protein structures into indexes [30]. These curves of ProtDex2 and TOPSCAN were adapted from Aung and Tan's report [30]. The precision percentage is plotted on the y-axis and the recall percentage is plotted on the x-axis.

The goal of SARST is to create an efficient database search method, information retrieval techniques that have been widely used in many database search and management fields were used to evaluate its accuracy. As shown in Figure 3, FAST was the most accurate method. SARST was the third most accurate, and had a higher accuracy when compared with YAKUSA, 3D-BLAST, TOPSCAN, BLAST, and ProtDex2, the former three of which are linear encoding methods.

### Implementation: Performance using different structural classes

The 108 query proteins from Aung [30] were composed of SCOP entries belonging to the four major classes with an average family size of ~80. To examine the performances of SARST using different structural classes, a measure known as "fallout" was calculated after the retrieval of 80 proteins.

Fallout=Number of irrelevant proteins retrievedTotal number of irrelevant proteins
 MathType@MTEF@5@5@+=feaafiart1ev1aaatCvAUfKttLearuWrP9MDH5MBPbIqV92AaeXatLxBI9gBaebbnrfifHhDYfgasaacH8akY=wiFfYdH8Gipec8Eeeu0xXdbba9frFj0=OqFfea0dXdd9vqai=hGuQ8kuc9pgc9s8qqaq=dirpe0xb9q8qiLsFr0=vr0=vr0dc8meaabaqaciaacaGaaeqabaqabeGadaaakeaacqqGgbGrcqqGHbqycqqGSbaBcqqGSbaBcqqGVbWBcqqG1bqDcqqG0baDcqGH9aqpdaWcaaqaaiabb6eaojabbwha1jabb2gaTjabbkgaIjabbwgaLjabbkhaYjabbccaGiabb+gaVjabbAgaMjabbccaGiabbMgaPjabbkhaYjabbkhaYjabbwgaLjabbYgaSjabbwgaLjabbAha2jabbggaHjabb6gaUjabbsha0jabbccaGiabbchaWjabbkhaYjabb+gaVjabbsha0jabbwgaLjabbMgaPjabb6gaUjabbohaZjabbccaGiabbkhaYjabbwgaLjabbsha0jabbkhaYjabbMgaPjabbwgaLjabbAha2jabbwgaLjabbsgaKbqaaiabbsfaujabb+gaVjabbsha0jabbggaHjabbYgaSjabbccaGiabb6gaUjabbwha1jabb2gaTjabbkgaIjabbwgaLjabbkhaYjabbccaGiabb+gaVjabbAgaMjabbccaGiabbMgaPjabbkhaYjabbkhaYjabbwgaLjabbYgaSjabbwgaLjabbAha2jabbggaHjabb6gaUjabbsha0jabbccaGiabbchaWjabbkhaYjabb+gaVjabbsha0jabbwgaLjabbMgaPjabb6gaUjabbohaZbaaaaa@97BB@

**Figure 4:**
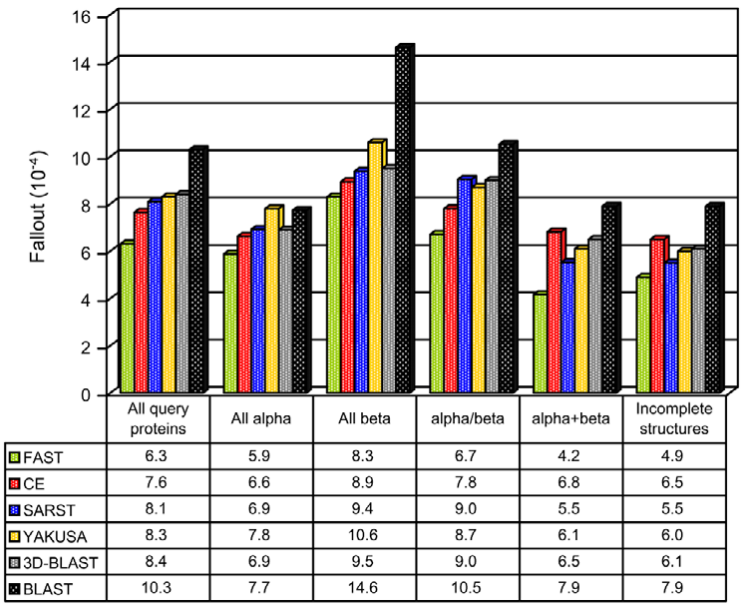
**Performances among different structural classes and proteins with incomplete structures**. The average fallouts after the retrieval of 80 proteins were calculated. Because fallout is a measure of the false positive rate, these data demonstrate that the performances of SARST are fairly even (i.e. no obvious bias) among the four major classes as compared to those of BLAST. The fallouts of SARST are generally lower than YAKUSA [21] and 3D-BLAST [22], both of which are protein structural similarity search tools with linear encoding methodologies. When tested with query proteins having incomplete local backbone structures, SARST outperforms other linear encoding methods and CE. The query proteins used in this experiment was set by Aung and Tan [30]; the subset of incomplete structures and the extent of incompleteness are listed in the supplementary materials [see Additional file 5].

Fallout is a measure of the false positive rate; it is the probability of retrieving an irrelevant protein [40]. Accordingly, an effective retrieval system will yield lower fallout. SARST generated lower fallout values when compared with recent linear encoding database search methods, YAKUSA and 3D-BLAST (Figure 4). The fallout rates of SARST are close to those of CE. Unlike BLAST, SARST and other structure-based algorithms, inclusive of linear encoding ones, had limited bias among the four structural classes.

### Performance on incomplete structures

Linear encoding methods may have a weakness in transforming structural information for proteins with incomplete backbone coordinates or missing residues [18], which constituted about one-fifth of Aung's query proteins [30] and the entire ASTRAL SCOP dataset. The fallout values of SARST for query proteins with incomplete structures were compared with those of several other methods. As illustrated in the right part of Figure 4, SARST generated fewer false positives than other linear encoding methods and, more interestingly, CE (see Additional file 5 for further information). These data indicate that SARST has achieved improved performance on incomplete structures.

### Effects of low sequence identities

To more precisely assess the efficiency of searching methods challenged with low sequence identities, a well-organized, non-redundant target database in which all query proteins have remote homologs is necessary. Since Aung's databases do not satisfy this purpose [30], we have generated a new database from the ASTRAL SCOP 1.69 dataset. Query proteins were selected from the ASTRAL 100% ID subset following these criteria: **(1) **belonging to the four major classes (all-*α*, all-*β*, *α*/*β *and *α*+*β*), (2) having a family size between 30 and 140 proteins, **(3) **sharing 10% or less sequence identities, **(4) **having at least two family members in the 10% ID subset, **(5) **having no missing residues or incomplete backbone coordinates, and **(6) **being able to reach 100% recall with all of the assessed tools. The proteins meeting criteria (1)–(5) were grouped according to the family classifications and ranked by their lengths; then, the median length from each family was chosen and tested for criterion (6). The 83 query proteins that met these criteria were subtracted from the original subset, yielding a target database of 24,337 proteins [see Additional file 3].

**Figure 5:**
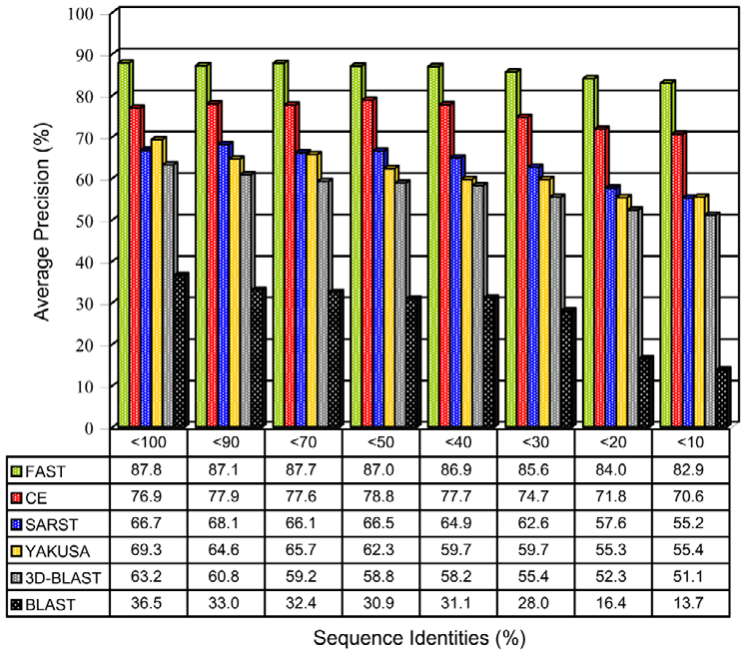
**Effects of sequence identities on the precision of several search methods**. The structure similarity search method, SARST, was able to detect remote homology with increased precisions compared with other linear encoding algorithms and the conventional amino acid sequence search method, BLAST. These data also show that there is still room left for the improvement of linear encoding methodology. Possible solutions are proposed in Discussion. The average precisions used in this figure were calculated at the representative 60% recall level.

Using this new target database, IR experiments were performed to examine the effects of low sequence identities. Various identity subsets of the target database were searched. As shown in Figure 5, the precision of SARST decreased as it encountered proteins with low sequences identities but was not as negatively affected as the precision of BLAST, which decreased substantially when the sequence identities fell below 30%. In comparison with recent linear encoding methods like YAKUSA and 3D-BLAST, the precision of SARST was generally improved. It could be observed that, when tested with these non-redundant datasets, the accuracy of linear encoding methods was substantially lower than geometric algorithms like FAST and CE. We propose that this is because of the unavoidable loss of structural information in the process of 3D to 1D transformation, a phenomena discussed in the latter part of this article.

### Reliability of searching results

For every retrieved structure, SARST provides not only a similarity score but an expectation value (E-value) to assess the significance of the score (see Discussion). A lower E-value correlates to a higher significance of the score. IR experiments were done to test the reliability of the E-value. As shown in Table 3, low E-values gave high precisions and low fallouts at both the superfamily and family levels. When E-values were below 10^-10^, for instance, the average precision was greater than 92% and the average fallout was lower than 0.04%. Thus, the rate of negative answers' being retrieved (as positives) was at most 0.04% by SARST in this particular database search. The reliability of the E-value lends greater significance to the structural, functional and even evolutionary relatedness information retrieved by SARST.

**Table 3:** Average recall and precision with various E-values

	Family level				Superfamily level		
			
E-value	Avg. recall (%)	Avg. precision (%)	Avg. fallout (10^-4^)		Avg. recall (%)	Avg. precision (%)	Avg. fallout (10^-4^)
		
1.0E-40	30.37	99.83	0.01		20.75	100.00	0.00
1.0E-35	34.19	99.79	0.03		23.34	100.00	0.00
1.0E-30	38.75	99.69	0.09		26.49	100.00	0.00
1.0E-25	44.21	99.00	0.31		31.18	100.00	0.00
1.0E-20	49.85	98.28	0.60		35.80	99.94	0.05
1.0E-15	58.40	97.52	1.11		41.75	99.64	0.24
1.0E-13	61.40	97.10	1.95		44.14	99.33	0.78
1.0E-10	67.98	92.71	4.00		49.99	96.34	2.59
1.0E-07	74.13	83.61	14.71		56.26	83.80	15.88
1.0E-05	78.49	71.00	46.80		61.83	68.26	57.58
1.0E-03	83.04	60.37	141.24		68.83	49.16	193.98
1.0E-01	89.22	48.39	429.86		77.66	32.66	648.34
1	91.60	46.19	687.41		82.03	27.92	1127.10
10	94.12	43.60	1101.13		86.94	25.91	1911.62
100	95.98	40.92	1680.02		91.36	24.85	3004.44
1000	97.63	38.50	2335.32		95.20	23.83	4468.79

### Normalization of SARST scores

According to our observations, larger proteins generated higher SARST scores, which did not always translate into smaller root mean square distances (RMSD) in the actual structural superimpositions. For this reason, in some situations, it would be better to normalize SARST scores. The same formula used to normalize FAST scores [6] was used to normalize SARST scores.

S˜=SM×N
 MathType@MTEF@5@5@+=feaafiart1ev1aaatCvAUfKttLearuWrP9MDH5MBPbIqV92AaeXatLxBI9gBaebbnrfifHhDYfgasaacH8akY=wiFfYdH8Gipec8Eeeu0xXdbba9frFj0=OqFfea0dXdd9vqai=hGuQ8kuc9pgc9s8qqaq=dirpe0xb9q8qiLsFr0=vr0=vr0dc8meaabaqaciaacaGaaeqabaqabeGadaaakeaacuWGtbWugaacaiabg2da9maalaaabaGaem4uamfabaWaaOaaaeaacqWGnbqtcqGHxdaTcqWGobGtaSqabaaaaaaa@34A9@

where *S *is the raw score and S˜
 MathType@MTEF@5@5@+=feaafiart1ev1aaatCvAUfKttLearuWrP9MDH5MBPbIqV92AaeXatLxBI9gBaebbnrfifHhDYfgasaacH8akY=wiFfYdH8Gipec8Eeeu0xXdbba9frFj0=OqFfea0dXdd9vqai=hGuQ8kuc9pgc9s8qqaq=dirpe0xb9q8qiLsFr0=vr0=vr0dc8meaabaqaciaacaGaaeqabaqabeGadaaakeaacuWGtbWugaacaaaa@2DEA@ is the normalized one. M and N are the RM string lengths for two proteins. The precision increased when the hit list was rearranged in a descending order according to the S˜
 MathType@MTEF@5@5@+=feaafiart1ev1aaatCvAUfKttLearuWrP9MDH5MBPbIqV92AaeXatLxBI9gBaebbnrfifHhDYfgasaacH8akY=wiFfYdH8Gipec8Eeeu0xXdbba9frFj0=OqFfea0dXdd9vqai=hGuQ8kuc9pgc9s8qqaq=dirpe0xb9q8qiLsFr0=vr0=vr0dc8meaabaqaciaacaGaaeqabaqabeGadaaakeaacuWGtbWugaacaaaa@2DEA@ value. For example, when SARST was run with Aung's database [30] under the recommended parameter settings (see Methods), the average precision increased from 84.1 to 86.3% after normalization.

The normalized scores were more sensitive to global structural similarities and thus more likely to retrieve SCOP family members, which were mainly clustered according to their overall structural similarities. However, local similarities, which measure the structural relatedness of substructures such as domains, are also important in many situations. Hence, the score normalization is adjustable by the user in the SARST web service.

### Distantly related homologs retrieved by SARST – two examples

We selected two pairs of proteins to demonstrate how SARST could detect remote homology from a large structure database. These protein pairs were retrieved from the ASTRAL SCOP 1.69 dataset. The coordinates of the positive positions aligned by RM strings were extracted to perform superimposition before calculation of their minimum RMSD.

**Figure 6:**
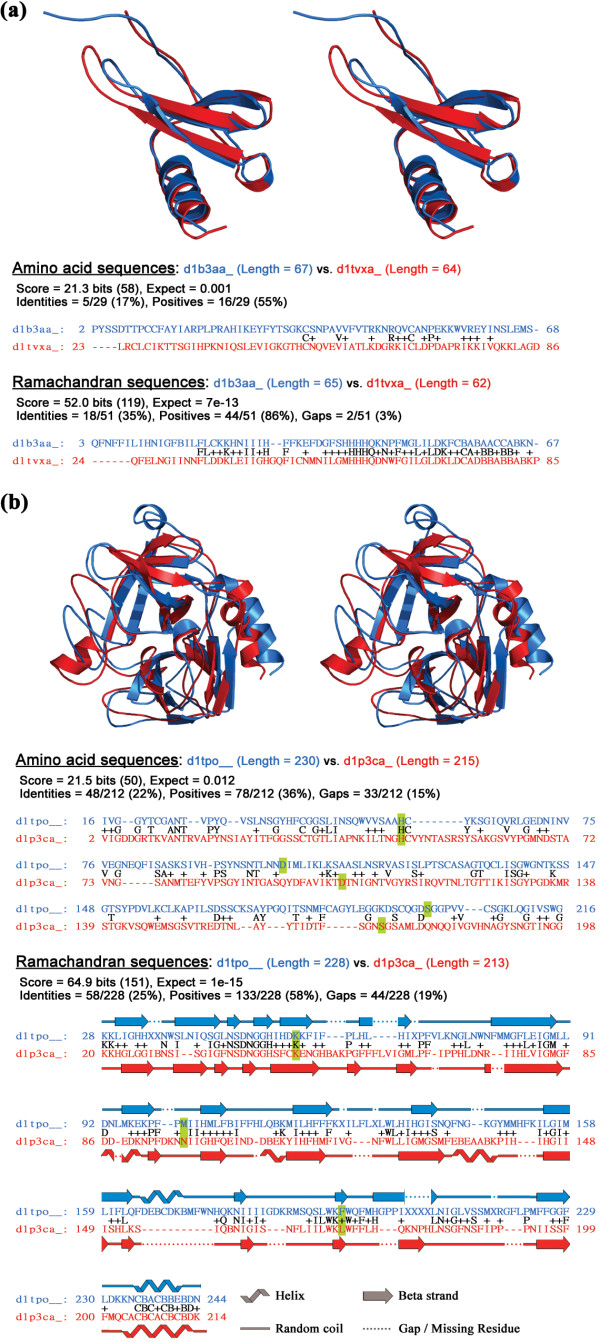
**Examples of distantly related proteins retrieved by SARST**. (a) The superimposed structures (cross-eye stereo view) of the interleukin 8-like chemokines from human, [SCOP:d1b3aa_] (blue) and [SCOP:d1tvxa_] (red). These two proteins have a low sequence identity while their structures are highly similar. The minimum RMSD calculated from positive positions of the Ramachandran sequence alignment is 1.68 Å. (b) Three-dimensional structures and the superimposition of [SCOP:d1tpo__] (blue) (SCOP sccs id: b.47.1.2), the trypsin from *Bos taurus*, and [SCOP:d1p3ca_] (red) (SCOP sccs id: b.47.1.1), a *Bacillus intermedius *glutamyl endopeptidase. These two proteases belong to different families but have similar structures. Although the amino acid sequence alignment fails to detect their functional similarities, the catalytic triad residues (highlighted in green) are well aligned by SARST. Their minimum RMSD is 4.17 Å, whereas their amino acid sequence identity is 22%. The secondary structural cartoons were generated by PROCHECK [54] and then modified with colors and gaps.

In the first example (Figure 6a), [SCOP:d1b3aa_] was the query protein and [SCOP:d1tvxa_] was one of its relevant retrievals. Both of these proteins are interleukin 8-like human chemokines. Their amino acid sequence identity was only 17.2% over a small alignment length (29 residues), whereas they were structurally very similar (minimum RMSD: 1.68 Å) with a much larger RM string alignment length (51 positions). This example indicates that SARST could successfully identify protein homologs sharing highly conserved 3D structures but low overall sequence homology (also seen in Figure 5). In the second example (Figure 6b), [SCOP:d1p3ca_], a *Bacillus intermedius *glutamyl endopeptidase, was a high score irrelevant retrieval of the query protein [SCOP:d1tpo__], trypsin from cow (*Bos taurus*). These two proteases exhibited only a 22% amino acid sequence identity. They had similar structures, and the catalytic triads were well aligned by SARST even though they belong to different families in the SCOP classification. There were several missing residues in the query protein, and there were major differences in length for some of the secondary structure elements (SSE), which would normally cause some failure to previous linear encoding methods [18]. SARST successfully identified the structural and functional similarities using suitable "X" scores and gap penalties. (Note that SARST is a database search tool that aims to rapidly distinguish high from low similarities but not to give optimum pairwise structural alignments. The RM sequence alignments shown in Figure 6 demonstrate how SARST works on protein homologs sharing low amino acid sequence identity but does not guarantee the best way to superimpose protein structures.)

## Discussion

### On speed

SARST [28] transformed structural information into text strings through the Ramachandran plot, and converted complex geometric superimposition problems into relatively simple sequence similarity search problems. Therefore, SARST compared favorably with conventional structure alignment search methods in terms of speed.

Because SARST uses a relatively simple scoring scheme and an optimized scoring matrix, it ran remarkably faster than previous linear encoding methods, like TOPSCAN, YAKUSA and 3D-BLAST. There are several structure similarity search tools that can run at impressively high speeds by searching databases stored with pre-analyzed structural information, such as ProtDex2. SARST was not only faster than ProtDex2 but also much more accurate.

Given reasonable thresholds and a single CPU, SARST could run more than two hundred thousand times faster than CE. In a multi-processor system, SARST can automatically distribute the calculation work and run even faster. For example, when we used 2 hyperthreading CPUs to run SARST, it executed over half a million times faster than CE, which could not support multiple processors unless it were run by multi-thread scripts programmed by the user. If SARST is run in a clustered environment, mpiBLAST [41-43] can be used as the search engine, and the increase in its running speed would be even more impressive.

### On accuracy

Although the current version of SARST could not match FAST in terms of precision, its accuracy is only slightly lower than that of CE. Additionally, SARST could achieve much higher precision than common IR-based tools, such as ProtDex2. In comparison with other linear encoding methods like TOPSCAN, YAKUSA and 3D-BLAST, the accuracy of SARST is improved. FAST is reportedly more accurate than CE [6]. SARST alone can serve as an efficient protein structural database search method; furthermore, a good web service can be developed if we combine SARST and FAST through a filter-and-refine strategy [31].

After examining the high score hits, we found that the irrelevant retrievals obtained by SARST were largely due to common substructures shared by proteins with different overall structures. In fact, because SARST uses BLAST (basic local alignment search tool) as the core search method, it is suitable for local structural alignment searches. We have suggested a method to normalize SARST scores, which could promote accuracy in the situation that users are more concerned about the overall structural similarities. Parameters such as gap penalties could be adjusted to achieve higher accuracy according to the needs of the user.

Alpha-helices are the most abundant form of regular secondary structure, and therefore the alpha helix-related codes inevitably have the highest occurrence in linear encoding methods [22]. Because there is high probability that two alpha helix-related codes could be aligned by chance, one may expect that SARST, as well as many other linear encoding methods, would produce more false positives in searching structural homologs for all-alpha proteins. However, our results indicated that SARST and other recent linear encoding algorithms had fairly even performance for different structural classes (Figure 4) in comparison with traditional sequence alignment method. In the case of SARST, which outperforms other linear encoding methods, this improvement may result from two factors. (1) The substitution matrices were generated with Henikoffs' algorithm [38], which calculates similarity scores as the logarithm of the odds (lod) ratio of the observed versus expected probabilities of every code pair. The most abundantly occurring helix-related RM code pairs thus do not have high lod scores, preventing the overweighting of helical SSEs. (2) The introduction of *T*_*N*_, the threshold of group size, into Ramachandran sequential transformation, resulted in fine dissections of the helix-like region of the RM plot. There were nine helix-like RM codes, e.g. ABCDETKVP, enabling SARST to detect minor structural differences between two helical SSEs, reducing the false positive rate.

### On improvements

Missing residues can reduce the performance and accuracy in protein structural similarity searches, as reported in the SA-Search linear encoding system [18]. SARST, however, uses "X" codes to represent missing residues and, given suitable X scores and optimum gap penalties, it suppresses the effects of structural incompleteness (Figure 4).

The precision of SARST was higher than TOPSCAN, YAKUSA and 3D-BLAST probably due to torsion angle properties. Torsion angles are too local to describe long-range residue-residue interactions and may be insufficient for the development of structure "alignment" methods; however, in developing "similarity search" methods through linear encoding, this regional property may have advantages. We hypothesize that linear encoding methods lose structural information in the transformation process, and thus the more information to be encoded the more likely it would be lost. As shown in Table 2 and Figure 3, SARST, encoding two-residue-long conformations by torsion angles, was faster and more accurate than YAKUSA, 3D-BAST and TOPSCAN. The YAKUSA algorithm uses alpha angle to convey four-residue-long interactions [21], 3D-BLAST uses alpha and kappa angles to describe five-residue-long backbone conformations [22], and, TOPSCAN considers even longer topological changes of secondary structural elements [17]. These results may imply that the "encoding ratio" has an inverse relationship to the range of interactions and may play a major role in protein structural linearization methodologies.

We plan to modify the SARST algorithm to preserve more structural information in the transformation process and to achieve higher accuracy. Future versions of SARST may consult the hidden Markov model and the methods of the Camproux-derived structural alphabet 27 [44,45]. About the alphabet size of Ramachandran codes, we had made many preliminary tests ranging from 13 to 23 prior to the choice of 23. It was found that, at least in this range, a larger alphabet size gave a higher precision; however, the performance of the current version of SARST is limited by its search engine such that a maximum of only 23 symbols can be used to compose RM sequences. We hypothesize that if more symbols could be used, the dissection of the Ramachandran plot would be finer, thereby increasing the accuracy.

### Significance of SARST score

After the database search, SARST produces a list of hits ordered by a score measuring the structural similarity. Additionally, SARST provides the statistically meaningful E-value to assess the significance of the score (*S*). The E-value is the number of different alignments with scores equivalent to or better than *S *that are expected to occur by chance in a database search [36,37,46]. Thus, lower E-values yield more significant scores. This statistical significance is transferable to structural relatedness and functional classifications. For instance, as shown in Table 3, the retrievals with E-values lower than 10^-25 ^almost all belong to the same family as the query protein (average precision > 99%; average fallout < 3.1 × 10^-5^). A score with an E-value lower than 10^-13 ^can be regarded to have a superfamily-level significance as the average precision at the superfamily level is higher than 99% and the average fallout is lower than 7.8 × 10^-5 ^under this E-value threshold. Thus, the chance that one retrieved protein with such a low E-value belongs to a different superfamily from the query is at most 7.8 × 10^-5 ^in this particular database search.

Proteins in the same SCOP family have a clear evolutionary relationship and those sharing the same superfamily most likely have a common evolutionary origin [47]. Automated procedures like Classification by Optimization (CO) [48] have been developed to link the Z-score, a measure of the statistical significance of the result relative to an alignment of random structures [4,5,49], to SCOP classifications and thereby predict protein evolutionary relationships, to which we hypothesize that the E-value provided by SARST is also transferable.

### Expected applications of SARST

The primary advantage of SARST is its speed. SARST provides a high search speed without substantially compromising the accuracy. Identification of distantly related protein homologs from a large structural database may prove difficult for sequence search methods or be time-consuming when using conventional structural alignment methods; however, SARST can accomplish this task within one second. In addition, this methodology is easy to implement, and multiple parameters can be adjusted by users to meet their research preferences. Moreover, the stand-alone version of SARST is written in Java and can run on many different platforms, turning a personal computer into an efficient instrument for a protein structural similarity searches.

Because of its high efficiency and portability, we hypothesize that SARST will be useful in automated and high-throughput functional annotations or predictions of the rapidly increasing protein structures produced by structural genomics researches. Because SARST describes protein structures as 1D strings, it can work together with multiple sequence alignment tools such as CLUSTAL W [50,51] to perform rapid structural, functional or evolutionary clustering of proteins. In addition, fold-recognition and backbone structure predication have used the one-dimensionalization of protein structures for years [19,20,24-27] and may be applicable fields for SARST.

## Conclusion

We have introduced a new protein structure similarity search method, SARST (Structural similarity search Aided by Ramachandran Sequential Transformation), which transforms 3D protein structures into 1D strings through a clustered Ramachandran map [28]. This technique uses a regenerative approach to produce improved substitution matrices and recruits classical sequence alignment search methods to perform structural similarity searches. As a hybrid, SARST combines the speed advantages of sequence-based methods and accuracy advantages of structural comparisons. Its precision is only slightly lower than CE, and SARST executes hundreds of thousand times faster, almost as rapid as BLAST. In addition, SARST provides E-values to assess the reliability of the retrieved information.

SARST can detect remote homology that escapes a typical amino acid sequence alignment search. Its performance among different structural classes is similar to that of CE, without the normal bias shown by BLAST. Compared with previous linear encoding methods, SARST suppresses the problems caused by structural incompleteness by utilizing "X" codes and major differences in SSEs between homologous structures by using suitable gap penalties: it also achieves higher search speed and precision.

The fact that most linear encoding methods could not match conventional structure alignment methods in accuracy indicates that linear encoding might not be the best solution to protein structural comparisons; however, SARST demonstrates that it still has the potential to develop efficient structural similarity search tools. Protein structural data is increasing exponentially; thus, we hypothesize that efficient, easily accessible and highly portable similarity search methods like SARST will be the basic tool for post-genomic era researches.

## Methods

The operating system was linux (Fedora Core 4) and, PHP (v.5.0.4) and Java 2 (v.1.4.2) were used to develop programs. The blast method described by Altschul *et al. *[36,37] was used as the SARST search engine. All structures presented in the figures were drawn using PyMol [52].

### Optimization of the search engine parameters

Because blastall (v.2.2.13) was recruited as the search engine, its parameter settings would affect the performance of SARST. Based on our early experience, the query sequence filter must be disabled (parameter setting: -F F) to achieve better search results. In addition, three other parameters were optimized: word size (W), gap-opening penalty (G), and gap-extension penalty (E).

There were two W values (2 and 3) allowed by blastall. We had used the small database developed by Aung [30] to determine their effects. It was found that the word size had limited effects on the precision of SARST, but the speed of SARST running under W = 3 was 3.4 times as rapid as that under W = 2. To meet speed requirements, size 3 was adopted.

At setting W = 3, the effects of all allowed combinations of G and E values were analyzed after the retrieval of 500 proteins. As shown in the additional material [see Additional file 4], SARST yielded the highest IR quality when G = 9 and E = 2, and it ran fastest when G = 25 and E = 2. Therefore, these are the recommended settings for SARST.

### Practical parameter settings for SARST

Generally speaking, a well-developed search tool, such as NCBI's BLAST [53], would offer many parameters freely adjustable by the user to satisfy individual research preferences; however, a set of default values should also be provided to meet the common needs of users and to ensure high performance. To determine the practical parameter settings of SARST, Aung's large database [30] was used to compare the precision and speed of SARST under various v (number of database sequences to show one-line descriptions) and e (expectation value, or E-value) thresholds. When the v threshold was 250, the average recall was over 80%; thus, higher values seemed unnecessary. When the E-value threshold was above 10^-7^, the average precision fell below 80%; thus, higher thresholds appeared impractical. As such, we suggest that the combination of v = 250 and e = 10^-7 ^would satisfy common needs. Running under these settings, the average recall of SARST was 76.0% and the average precision was 84.1%.

### Assessment of speed and precision

Among the tools assessed in this report, the stand-alone version of CE and FAST could only perform pairwise comparisons; hence, the database searches were achieved using numerous pairwise comparisons with script programs. However, to make fair assessments only the actual running times were considered. The calculation times for parsing and sorting the results were omitted. The time consumed in parsing the outcomes of BLAST, ProtDex2, YAKUSA, 3D-BLAST and SARST was also omitted.

CE was good at local alignment, and therefore its output might contain many pairwise alignments of polypeptide fragments [5]. In such cases, the alignment with the greatest length was selected as the final result.

FAST was designed to align two single polypeptide chains [6]. Because many SCOP domains were composed of multiple fragments from different chains, they would cause FAST to function improperly. Thus, before any PDB file of SCOP domains was entered, it had to be "unified" first – all the chain IDs were changed to "A" regardless of the original labels, and all the residues were re-numbered consecutively.

## Authors' contributions

WCL performed the clustering of the Ramachandran map, carried out the information retrieval experiments and participated in the design of the study. PJH participated in the design of the study and the production of scoring matrices. CHC participated in the development of the web service. PCL has conceived and coordinated the study. All authors contributed to writing the manuscript.

## Supplementary Material

Additional file 1 The training set. A list of the SCOP domains in the training set. Click here for file

Additional file 2 Performances of SARST scoring matrices. The F-measure and running time for SARST using different scoring matrices. Click here for file

Additional file 3 The query and target proteins. A list of the 83 query proteins and the large target database containing 24,337 proteins collected from the ASTRAL SCOP 1.69 100% identity subset. Click here for file

Additional file 4 Effects of gap penalties. The average recall, precision, F-measure and running time for SARST under various gap penalties. Click here for file

Additional file 5 Query proteins with incomplete structures. A subset of Aung and Tan's query proteins [30] that contain missing residues and/or incomplete backbone structures. The extent of structural incompleteness is provided, too. Click here for file
